# Annotations

**Published:** 1899-12-23

**Authors:** 


### Annotations.

### The Late Sir Richard Thorne Thorne, K.C.B.

We greatly regret to have to record the death of Sir Richard Thorne Thorne, K.C.B., who has for some years been principal medical officer of the Local Government Board, and has on many important occasions been consulted by her Majesty's Government in regard to international sanitary measures. About 30 years ago he became attached to the medical department of the Privy Council, which was afterwards transferred to the Local Government Board, and ultimately became its head, succeeding the late Sir George Buchanan. Great, however, as was the work done by Sir Richard Thorne Thorne in direct connection with his office in organising the sanitary defences of the country, in the direction of measures for the investigation and control of epidemics, and in raising the standard of sanitation demanded all over the country, much of the work which brought him more particularly before the public eye was what he did in relation to the various international sanitary conferences to which he was sent as a delegate by the Government. In these conferences, which met successively at Rome, Venice, Dresden, and Paris, he took a leading part, and by his intimate knowledge of the subject, his courtesy, and his unflinching firmness in the matter of first principles there can be no doubt that he became mainly instrumental in convincing the representatives of other nations of the futility of quarantine. In this way he became a benefactor to the world. Perhaps Sir Richard's last public utterances were at the recent meeting of the General Medical Council, when he strongly protested against the idea that medical attendance was a necessity for a healthy woman in normal labour. Sir Richard died, we believe, quite suddenly from embolism, arising from some inflamed varicose veins.

### Contagious Disease among our Soldiers.

It is much to be feared that, as has happened again and again in our smaller wars, so also in South Africa, England will have to pay the penalty which naturally follows from her *laisser faire* method of dealing with those contagious diseases which are so prevalent in most of our Indian stations, and do such damage to our army as a fighting force. At immense expense we every year send out to India hundreds of young men, whom we maintain there in as high a state as possible of efficiency and of animal vigour. Yet, with strange perversity, notwithstanding the elaborate precautions taken to protect their health and to shield them from infection of one sort and another, there is one class of infection before which we stand helpless, preferring to see our men diseased and their fighting power impaired rather than even appear to sanction vice or to interfere with its "natural punishment." These young fellows, who must of necessity in most cases be unmarried, are left exposed to every kind of temptation; they have plenty of time on hand, they have a fair amount of liberty, indulgence in vice is cheap, and unfortunately unlimited opportunities are provided for the satisfaction of every lust. Knowing then, as we do, that if once they give way to the wiles of the temptress they will become diseased, and be rendered useless for the very purpose for which they were enlisted, what steps do we take to prevent infected women from plying their trade and spreading their vile diseases among our troops? None. We stand aside and, in the name of virtue and respectability, allow everything that is most vicious and disreputable to go its own way. Can we wonder, then, that these young fellows fall before the native women and their touts, that venereal disease becomes the great and predominant cause of sickness among our troops, and that when we embark upon a war, the base hospitals become occupied by men who have broken down before ever they have arrived at the front at all? It is an absurd position, and it is the result of an absurd misuse of the national conscience. Typhoid fever is a filth disease. Are we, then, encouraging, or, as the phrase goes, "legalising" filth, when we isolate those suffering from its effects? Yet such is the plea upon which resistance is opposed to every effort to bring this vile trade under sanitary control. Those who have for so long stood in the way of all attempts to prevent diseased women from plying this trade have been responsible for the existence of a large amount of preventable sickness both in the army and among the civil population. Now they must, in addition, be held responsible for the disagreeable fact that a not inconsiderable number of our soldiers, especially those arriving from India, have turned out to be unfit for the stress of war, and have served only to fill the beds intended for their wounded comrades.

### The Sick and Wounded.

An outcry is being raised about the sick and wounded being brought home to face the inclement weather of an English winter, instead of being left to recover in what "A Hospital Physician," writing in the *Times*, describes as "the bright sunshine of a South African spring." But we think with Surgeon-General Hamilton that those who are able to bear the transport are better at home than in a country where everything is in a state of turmoil and disturbance, and the climate of which, however useful in the treatment of consumption in consequence of its permitting patients to live a large part of their time in the open, does not offer many advantages to those suffering from ordinary sickness. A South African spring is a short affair, besides it is now over. In those parts of the country in which the comforts of life are easily obtainable the climate is very relaxing at this time of year, while in the more elevated regions the life is of necessity very rough. Good food and reasonably good cooking are points of great importance during the progress of convalescence, and, whatever may have been the experience of wealthy visitors, all who have lived there agree that in both these particulars the higher and more healthy regions in South Africa are lamentably deficient. After all, these unfortunate sufferers will have to come home some time, and if they are now fit for the transit why not make it while vessels are abundant and ship room plentiful, rather than later on, in the midst of the crowd when the current of traffic sets in homewards? Nor must we remain oblivious of the sentiment of "Home, Sweet Home." Let them return then, and let a grateful country do all that is possible to relieve the distress of their sad home-coming.

